# Effect of Thermal and Vibration Changes on Automated External Defibrillator Circuit Boards: Finite Element Analysis Study

**DOI:** 10.2196/53208

**Published:** 2025-08-19

**Authors:** Saidi Olayinka Olalere

**Affiliations:** 1 Department of Mechanical Engineering, Georgia Southern University, 1332 Southern Drive, Statesboro, GA, 30458, United States, 1 9124784636

**Keywords:** circuit board, automated external defibrillator, heart, cardiology, vibration, thermal changes, medical devices

## Abstract

**Background:**

An automated external defibrillator (AED) is a device that is used to prevent sudden death by delivering an electrical shock to restore the heart rhythm when experiencing cardiac arrest.

**Objective:**

This study was performed to analyze the vibration and thermal changes experienced by an AED medical device when exposed to shocks caused by patients’ reactions, vibrations from mobile and air ambulances, and heat changes due to the battery component on the circuit board.

**Methods:**

Basically, AED is made from plastic, with the external parts containing the display, buttons, pad socket, and speaker, while the internal part entails the circuit boards comprising components such as resistors, capacitors, inductors, and integrated circuits, among others. In this study, the AED was modeled with the Ansys Workbench 2020 and calibrated based on static and dynamic loading to verify the static displacement and determine the first set of five frequencies obtained based on the unprestressed conditions.

**Results:**

Using the prestressed analysis with modifications, the next set of frequencies was obtained with an error margin of 0.0003% between each frequency. The modeled circuit board was used to examine the vibration and dynamic analysis for the rigid board. Similarly, thermal analysis was conducted on the modeled circuit board with the battery serving as the heat source. The rate of dissipation of heat around the board and its effect on the circuit components was evaluated.

**Conclusions:**

The modeled circuit board was reinforced with more support structures to mitigate the deformation effect. The deformation peaked at 33.172 mm, with minimum deformation at the edges of the board. Components with greater height, such as capacitors, experienced more pronounced deformation. Therefore, it is suggested that flat capacitors of lesser height would be suitable for future designs. Additionally, significant heat dissipation from the battery suggests a need for better dissipation pathways.

## Introduction

### Background

The automated external defibrillator (AED) is a lifesaving device designed to assist individuals experiencing sudden and life-threatening cardiac arrest caused by arrhythmias such as ventricular fibrillation or pulseless ventricular tachycardia. It functions by analyzing the heart rhythm and delivering an electrical shock to reverberate the heart and restore heart rhythm. The AED is made of plastic and has external and internal parts; the external part contains the pad expiration window, latch, status indicator, battery compartment, battery, electrode holder, color display, manual override buttons, shock button, pad or electrode socket, speaker, and infrared port. The internal parts include the mainboard, display board, speaker, display, shock discharge capacitor, and beeper speaker.

Sudden cardiac arrest is a leading cause of over 350,000 deaths in the United States. The average response time for a first responder is 8‐12 minutes, and for every minute of delay, the survival rate drops by approximately 10% [1]. The availability of AEDs significantly reduces this response time, underlining its importance in emergency situations.

The AED is a user-friendly instrument that can be operated without the need for specialized training. It is strategically placed in public areas to ensure easy access in the event of an emergency or sudden cardiac arrest. The success of the AEDs is evident in the Chicago Heart Start program, where 22 individuals in cardiac arrhythmia were treated, resulting in 11 successful recoveries. Notably, 6 of these successful treatments were administered by bystanders with no prior AED training.

The AED’s circuit board, crucial for its analysis, is constructed from Epoxy FR-4 and measures 254 mm in length, 216 mm in width, and 0.5 mm in thickness. The board is equipped with various components, including a capacitor, microcontroller, flash memory, analog-digital converter, field-programmable gate array (FPGA), processor, audio controller, and indicator. These components, along with others, form the basis of a finite element analysis (FEA) model, which is established based on the board’s design specification and components. The governing equation for the experiment is:


$${\text{Mx}}^{\prime \prime }+{\text{Cx}}^{\prime }+\text{Kx}=\text{F(t)}$$

where M, C, and K represent the mass, damping, and stiffness matrices, respectively.

The goal of the study was to analyze the effect of vibration and thermal experience on the AED, based on its operation. The model underwent various forms of static and dynamic testing of the modeled circuit board. Based on the number and position of the support configurations, the dynamic and vibration properties were analyzed for the modeled circuit board and the rigid board.

This study would assist in obtaining reliable results when the defibrillator is used in mobile ambulances, which experience vibration and road bumping. The AED has been a lifesaving device that is used in conjunction with cardiopulmonary resuscitation (CPR). The results from AED are important as they influence when CPR should be continued or stopped.

Finite element analysis was used for analysis and the model was designed using the Ansys Workbench for the circuit board, which contained integrated circuits. Static and dynamic tests were conducted on the model to determine the bending and damping effects, respectively.

### Literature Review

Deformation experienced in electronic components is classified as vibration, shock, and thermal failure. The AED circuit board is a device that experiences failure, which can impact the results obtained during the resuscitation of individuals experiencing cardiac arrest. Generally, most electronic circuit boards experience random vibration rather than ordinary vibration, due to external factors within the vibration environment. Most research on electronics is based on high-cycle fatigue to predict the fatigue life of components experiencing sinusoidal vibration. Fatigue failure under sinusoidal vibration loading has been analyzed by comparing results from vibration failure tests, FEA, and theoretical testing [2].

FEA modeling of the vibration of a printed circuit board (PCB) using rigid boundary conditions has been performed, comparing the modeling results against tests conducted using rigid fixtures to identify the PCB dynamic properties such as natural frequencies [3].

For random vibration fatigue, the circuit board research has extended to soldering by predicting the fatigue life when subjected to random excitation through vibration loading [4]. An experimentally validated vibration fatigue damage model for plastic ball grid array solder joint assemblies was developed by Wu [5] to calculate strain and solder joint survival using a three-band technique.

According to a study by Jadhav et al [6], a virtual simulation method was introduced for onboard charger assemblies using Noise, Vibration, and Harshness analysis. The onboard charger’s PCB components’ had a first natural frequency of 206 Hz, more significant than the testing’s working frequency range. Therefore, resonance effects on PCB components were found to be negligible. Subsequently, frequency response analysis showed that the produced stresses on the onboard charger were minimal. The acceptance criteria confirm the induced von Mises stresses, which fall within acceptable bounds.

As PCB technology becomes more sophisticated, it must balance both electrical and thermomechanical requirements. This study assessed the effects of the constituent layer qualities on the PCB stack-up properties. In the form of cured prepreg, five new FR-4 materials were examined, and thermomechanical parameters, including Young’s modulus, glass transition temperature, and coefficient of thermal expansion in the X/Y/Z planes, were measured. The results indicated that as prepreg’s glass fiber density rises, the X/Y coefficient of thermal expansion drops, and Young’s modulus increases. These values can vary by up to 50%. A reflow temperature of 250‐260 °C was ideal for lead-free solder; this will minimize the warpage and coefficient of thermal expansion mismatch during processing while increasing reliability. The selection and construction of PCBs with various glass fiber styles, form factors, and dimensions were based on promising FR-4 material options with greater glass transition temperature, reduced coefficient of thermal expansion, and optimized Young’s modulus. Prepreg characteristics enhance warpage performance and final PCB properties, regardless of PCB design. While the stack-up design decision mainly influences the PCB’s X/Y coefficient of thermal expansion, the PCB’s glass transition temperature and Z-coefficient of thermal expansion heavily depend on the resin material’s inherent qualities. Both prepreg characteristics and stack-up design decisions impact PCB modulus, as modulus is influenced by copper concentration and distribution. It is recognized that this relationship between prepreg characteristics and PCB stack-up can improve the understanding of printed circuit board design to achieve higher dependability [7].

In helicopter emergency medical services, a cabin that can swiftly and efficiently transport patients to a hospital is crucial. The quality and safety of the service may be affected by the vibration that patients and crew members experience during transportation, as a medical team uses life-support equipment to maintain the patient’s health. However, an incorrect assessment of vibratory level and exposure may result from the airframe’s bare dynamical response. The crew, patients, and medical supplies, through interfaces like seats, handles, stretchers, and flexible supports, dynamically interact with the helicopter. Therefore, to create a low-vibration helicopter emergency and medical service vehicle, the coupled helicopter-interface-subject system must be the subject of thorough numerical analysis. It should be possible to run the analysis effectively and efficiently across many possible configurations to achieve optimal positioning. Formulating high-fidelity rotorcraft aeroservoelasticity, connecting additional dynamical systems that represent the dynamics of humans and equipment, and calculating the vibration performance of the resulting models should all be possible with a viable tool. An efficient method for assessing the vibratory performance of medical helicopters was presented. The strategy is demonstrated on a medium-sized helicopter by adding dynamical models of a human resting on a seat, a recumbent person lying on a stretcher, and medical equipment mounted on flexible supports at its ends [8].

The study by Oon et al [9] investigated the warpage of both, a minor PCB with only one side and a large PCB with multiple layers using Shadow Moiré measurement and FEA. Primarily due to the absence of an initial warpage, simulation results from both printed circuit boards are lower than experimental results. At temperatures below the sample’s glass transition temperature, the disparity is reduced to 24% for the single-sided, small PCB and 15% for the multilayer, large PCB, respectively. The PCB conduct could not be reflected in the reproduction at the above glass transition temperature. Their findings demonstrate that the simplified model based on the copper content of each layer can be used to estimate the sample’s warpage for the multilayer, large PCB. When the copper content of each layer in the sample is increased to 100%, it is estimated using the same method that the PCB warpage would increase by 25%. In contrast, halving the copper content of each copper layer results in a 21% decrease in warpage.

The study by Yun et al [10] is a comprehensive examination of how well AEDs function during transportation in a moving ambulance. The researchers aimed to determine whether the motion of an ambulance affected the AED’s ability to detect and treat cardiac arrhythmias accurately. The study found that the performance of AEDs was reliable even while in motion, though certain variables, such as speed and road conditions, may affect their efficiency. This thorough research instills confidence in the reliable use of AEDs in emergency medical services during transport.

The study by Wang et al [11] explored the challenges of managing vibration levels in life-support systems used in medical helicopters. Their study focused on how excessive vibrations during air transport can affect patient care and the functionality of medical devices, such as ventilators and defibrillators. Using advanced simulation techniques, the researchers modeled how vibrations from the helicopter’s frame are transmitted to onboard equipment and patient positioning systems. They identified optimal configurations to reduce mechanical stress on life-support systems, ensuring improved patient stability and care during transport. The findings highlight the importance of minimizing vibrations to enhance the safety of medical air evacuations.

Recent advancements in random vibration fatigue research, especially in AEDs, have seen significant progress with enhanced FEA methods [12], including improved fatigue life predictions for solder joints within PCBs by simulating real-world vibrational conditions like those experienced in ambulances. Their study introduced advanced algorithms to represent random excitation forces better, by improving fatigue life predictions’ reliability. By refining input variables, their study enabled more accurate testing and earlier detection of potential failure points, thus enhancing the durability and safety of critical medical devices such as AEDs in unpredictable environments.

The studies by Caffrey et al, Chen et al, and Jespersen et al [13-15] investigated the functionality and reliability of AEDs in critical life-saving situations but from different perspectives [15]. Focusing on the operational status of AEDs registered for public use revealed that many AEDs were nonfunctional when needed for out-of-hospital cardiac arrests (OHCA), due to issues such as dead batteries or missing components, underscoring the importance of regular maintenance. Similarly, Chen et al [14] further evaluated the performance of AEDs under extreme environmental conditions, such as high altitudes and fluctuating temperatures, and found that these conditions lead to decreased battery life and impaired shock delivery efficiency. This study highlights the need for innovation in AED technology, mainly to ensure functionality in challenging environments, such as mountainous or cold-weather regions. Together, these studies emphasize the critical need for regular AED maintenance and design improvements to ensure reliability in varied emergency conditions, which is vital for improving survival rates in OHCAs.

Olalere and Choi [16] explored innovative voltage regulation techniques to improve the performance of electrosurgical devices, particularly of the hot snare polypectomy tool. Their work aligns with this study’s investigation into circuit board deformation in medical devices. The voltage control techniques developed by them helped optimize energy output during surgical procedures, thereby reducing thermal damage to surrounding tissues and improving patient outcomes. This study provides significant context for understanding how optimized electronic control systems can enhance the durability and functionality of medical devices, especially under stress or thermal conditions, as seen in the AED analysis. This research is directly relevant to ongoing work on improving device reliability in adverse conditions, such as high-frequency vibration environments and extreme thermal variations. Their focus on energy modulation parallels the need for efficient heat dissipation and improved circuit board resilience, which are key concerns in designing and optimizing critical medical equipment like AEDs.

Studies by Jonsson et al and Sarkisian et al [17,18] emphasize the critical role of AEDs in out-of-hospital cardiac arrest situations but from complementary angles. They highlighted the significant survival benefits of integrating AEDs into first responder systems, especially in public spaces. Their findings reveal that timely access to AEDs, typically within the first few minutes of cardiac arrest, drastically improves survival outcomes. Integrating technology, such as real-time location services, enhances the ability of first responders to locate and use AEDs more effectively, leading to faster defibrillation times and better resuscitation outcomes. This study underscores the importance of placing AEDs in high-traffic areas to maximize accessibility and efficacy [18]. Further examination of the effectiveness of AED deployment in different public and private settings revealed disparities in AED availability, with urban public areas being better equipped than residential locations, where most OHCA incidents occur. They emphasize the necessity for strategic AED placement and increased accessibility, particularly in residential and low-traffic areas, to close the gap in survival rates. Both studies converge on the idea that improved AED access and integration with first responder systems are essential for enhancing OHCA outcomes, primarily through strategic placement and the use of technology.

Several studies have explored the integration of drones in delivering AEDs to OHCA locations [19-21]. They focused on the experiences of dispatcher nurses managing these drone deployments, highlighting benefits such as faster AED delivery in rural areas, while also noting challenges such as synchronizing drone arrival with bystander readiness [20]. The authors emphasize regulatory, technical, and logistical barriers in coordinating drone technology with emergency medical services (EMS), identifying the need for standardized protocols [20]. Their study also notices that countries such as Sweden and Canada have made more significant progress in integrating drones into emergency systems than the United States. Schierbeck et al [21] have evaluated the effectiveness of drone-delivered AEDs in Sweden and showed that drones consistently arrive faster than ambulances in real-life OHCA scenarios. However, they also highlighted operational limitations such as weather conditions. These studies underscore the potential effect on the AED’s performance and efficiency in improving emergency response, particularly in rural or hard-to-reach areas. However, they also call for greater coordination, infrastructure improvements, and policy development to maximize their impact.

Several studies have collectively examined different strategies to improve defibrillation outcomes in OHCA locations [22-24]. They compared the effectiveness of on-site bystanders performing defibrillation with dispatched volunteer responders, highlighting that while on-site bystanders provide more immediate aid, dispatched responders often have better training and equipment. They suggest a hybrid approach where bystanders initiate CPR and responders handle defibrillation, increasing survival chances [23]. They also focus on the challenges of increasing defibrillation rates in home-based OHCAs, noting the lack of access to AEDs and limited public awareness as key barriers. Their recommendations include expanding AED distribution in residential areas and increasing public training [24]. An analysis of the effectiveness of volunteer responder systems in rural and urban areas found that volunteers significantly improved response times and survival rates in less densely populated areas, filling critical gaps where traditional emergency services may face delays. These studies underscore the need for integrated strategies, combining public training, AED distribution, and volunteer responders to improve survival rates across different geographic settings.

## Methods

### Model Preparation

The modeled circuit board components used in this study include capacitors, microcontrollers, flash memory, analog-digital converters, FPGAs, processors, audio controllers, batteries, and transistors.

The AED is a model comprising different components for analysis. The baseboard measures 254 mm × 216 mm.

The capacitors are cylindrical, with a length of 40 mm and a diameter of 35 mm. The microcontroller is 10 mm × 10 mm × 1.4 mm. The battery design is 34.5 mm in length and 17 mm in diameter.

The materials used in the components along with their respective Young’s modulus and Poisson ratio are presented in Table 1.

**Table 1.** Numerical constant of component.

Component	Material	Young’s modulus (GPa)	Poisson ratio	Thermal conductivity (W/mK)
Board	FR4 epoxy	24	0.118	0.81
Capacitor	Tantalum	175	0.34	54.4
Microcontroller	Copper	117	0.34	385
Flash memory	Polystyrene	3250	0.34	0.033
Analog digital converter	Silicon	140	0.275	150
FPGA^a^	Silicon	140	0.275	150
Processor	Silicon	140	0.275	150
Audio controller	Copper	117	0.34	385
Battery	Lithium	3.17E-05	0.355	5.4
Transistor	Silicon	140	0.275	150

a FPGA: field-programmable gate array.

**Figure 1.**
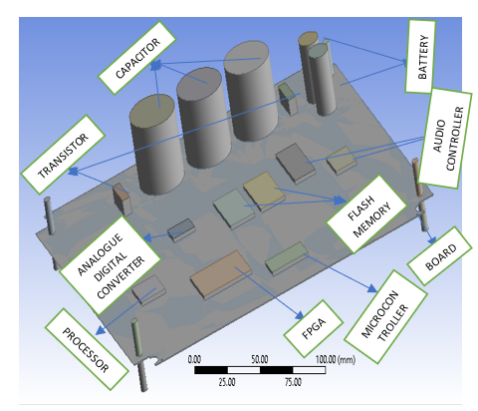
Model of the circuit board. FPGA: field-programmable gate array.

The modeled AED, developed using Ansys 2020 through Workbench, is a testament to our thorough approach to design. The model circuit board, designed based on the specific dimensions of the board and its components, further underscores our commitment to precision. Four fixed supports for the AED were fixed to the plastic casing of the AED, ensuring stability and reliability. The FEA, a powerful tool for deformation analysis and thermal effect assessment, was used to conduct a comprehensive analysis of the circuit board and its components, providing reassurance about the robustness of our design. The FEA model, a visual representation of our thorough analysis, is presented in Figure 1. The boundary condition was set at the four edges of the modeled circuit board, which are fixed as rigid bodies, mirroring a typical AED.

### Mesh Selection

**Figure 2.**
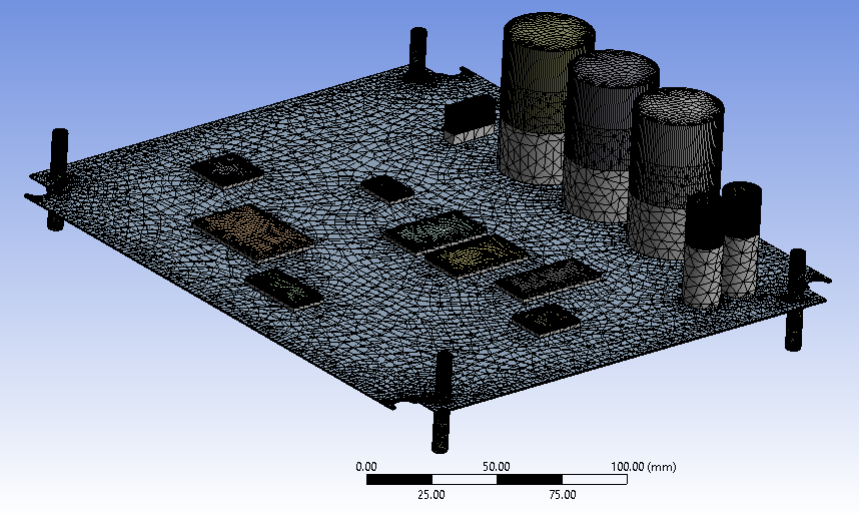
The mesh of the circuit board.

The modeled circuit board was meshed to verify the stress discontinuity of the member components attached to the board. The mesh model produces 90,371 nodes and 59,671 elements from program-controlled order (Figure 2).

### Ethical Considerations

This study did not require ethics board approval, as it did not involve human participants, identifiable personal data, or biological materials. No intervention or interaction with individuals occurred, and all data analyzed were obtained from simulation analysis. Therefore, an application to an institutional review board was not necessary, in accordance with policies and national regulations.

## Results

### Deformation Analysis for 4-Member Support

**Figure 3.**
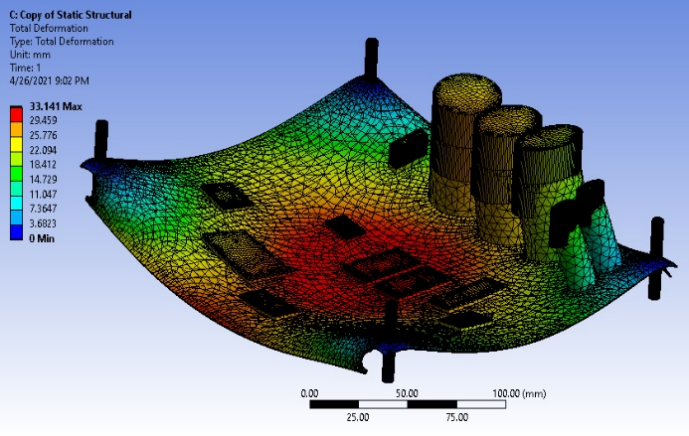
Static structural deformation for 4-member support.

The circuit board undergoes different deformation at different parts of the board. The maximum deformation is in the middle of the board, at a peak of 33.141 mm. Likewise, the entire board experiences edge bending, as shown in Figure 3. Taller components deform faster, causing damage to the circuit board.

The rotational deformation, measured with precision, is at its largest at the z-axis, with transverse at a 10% rate before converging at 60%. The x-axis shows a precise deformation at 10%‐60% of the time series. The y-axis deformation, also measured with precision, ranges between 10%‐80% of the time series before finally converging, as shown in Figure 3.

**Figure 4.**
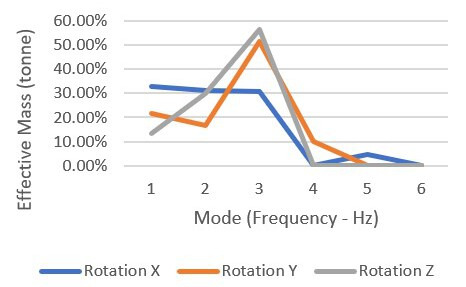
Effective mass to frequency for 4-member support.

According to Table 2 and Figure 4, approximately more than 30% of the effective mass contributed to the mode in the X direction. In comparison, 50% and 55% of effective masses contributed to the Y and Z directions, respectively.

**Table 2.** Mass participation for 4-member support.

Mode	Rotation X	Rotation Y	Rotation Z
1	33.669	15.71	21.22
2	3.20E+01	1.21E+01	4.72E+01
3	3.18E+01	3.71E+01	8.91E+01
4	1.61E-01	7.13E+00	1.33E-01
5	4.98E+00	9.73E-02	7.10E-03
6	2.18E-03	3.70E-03	4.09E-03
Overall	102.6497	72.17238	157.6688

Our research on the structural fatigue of the circuit board has yielded clear and important findings. We found that the center of the board, with less support than the four edges, experiences more visible deformation. This understanding is crucial for the development of more robust electronic components.

**Figure 5.**
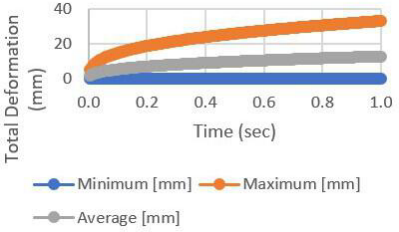
The total deformation rate of the circuit board.

Figure 5 shows the rate of deformation at an increasing level, which shows the time-to-failure for the specific components, such as resistors and capacitors, during the vibration test.

**Figure 6.**
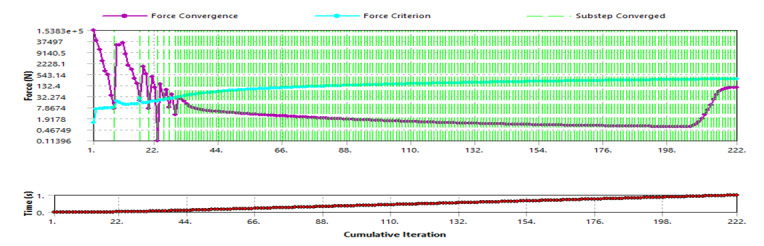
Force convergence.

For the force convergence in Figure 6, the substep converged towards the iteration end point. The convergence experienced longer iterations, allowing for the load to be evenly distributed, as seen by the substep. At 20% of the loading, normal stiffness was lowered to improve the analysis results.

**Figure 7.**
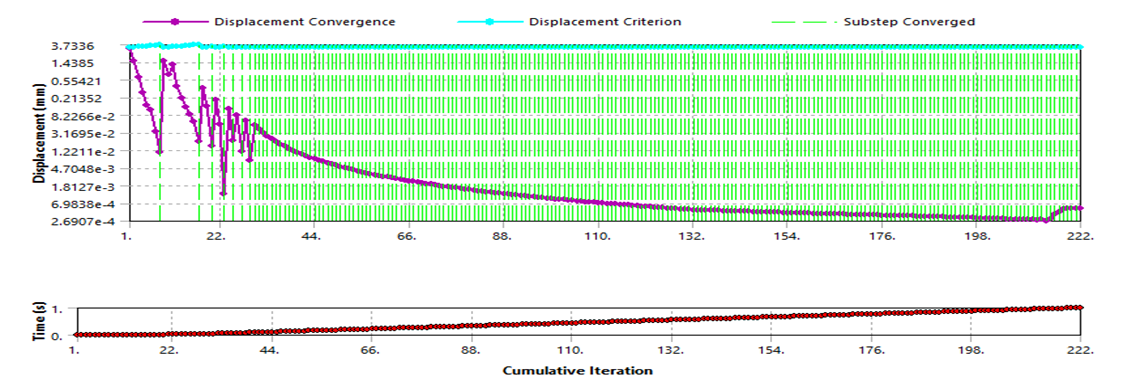
Displacement divergence.

From the displacement convergence in Figure 7, the standard stiffness was maintained towards the analysis’s tail end, indicating a consistent and accurate deformation rate from the 15% iteration.

**Figure 8.**
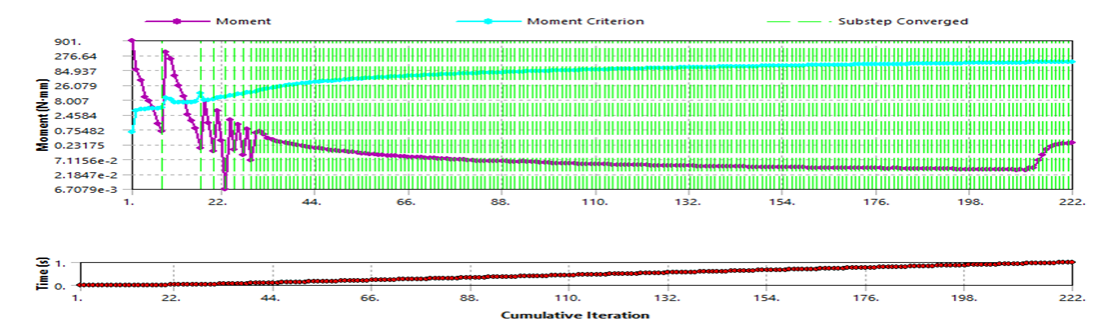
Moment convergence.

The moment of convergence in Figure 8, with the mesh refinement leading to node increment, demonstrates a uniformly converging analysis, ensuring the reliability of the results.

**Figure 9.**
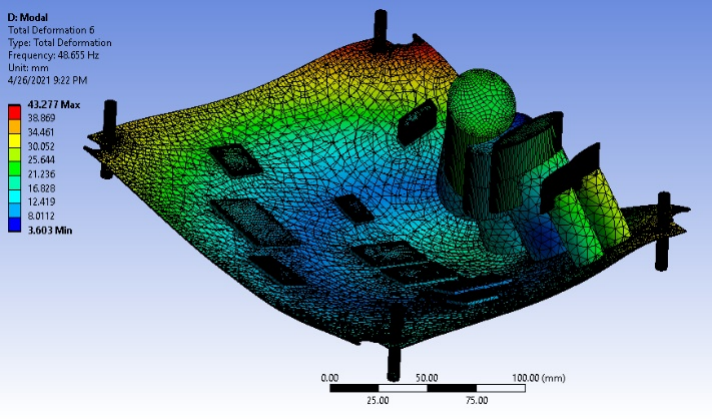
Unprestresses modal total deformation.

Figure 9 shows the deformation for an unprestressed condition. The deformation, which was more noticeable at the location of the capacitors, led to a significant distortion in the circuit board’s shape. This distortion could potentially affect the performance of the circuit board, highlighting the importance of considering deformation in the design process.

**Figure 10.**
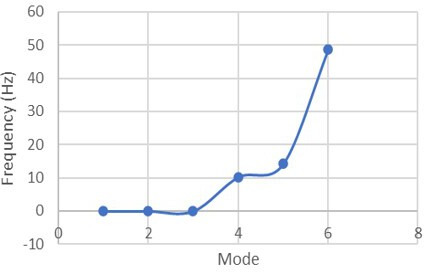
Unprestressed modal analysis.

The modal analysis used to investigate the vibration on the circuit board is used to evaluate the natural frequencies, as shown in Figure 10.

**Figure 11.**
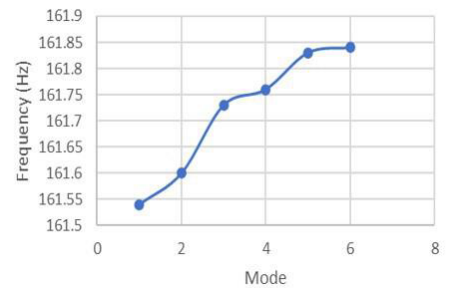
Prestressed modal analysis.

The prestressed analysis assists in improving the results obtained in the unprestressed process by modifying the stiffness to reduce the natural frequency inadequacies. This provides better and improved results for the simulation as the analysis was refined to give better frequencies as against the unprestressed, as seen in Figure 11.

### Deformation Analysis for 8-Member Support

**Figure 12.**
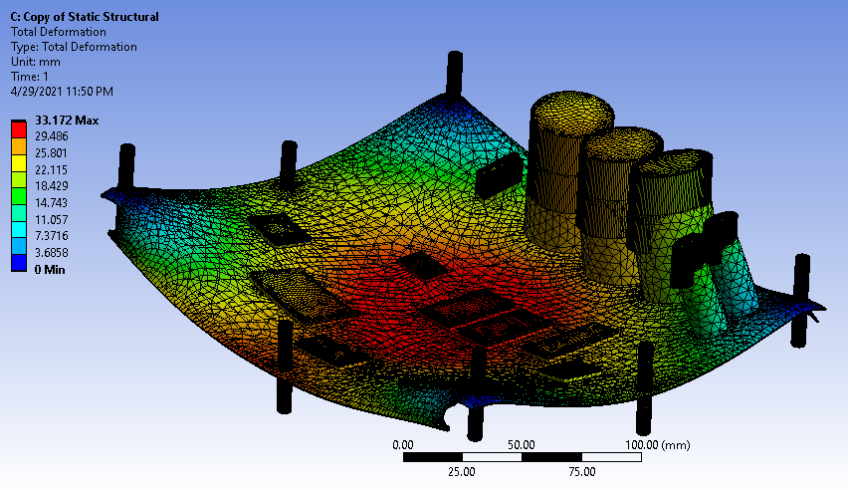
Static structural deformation for 8-member support.

The modeled circuit board was reinforced with more support members to improve its deformation effect. The deformation peaked at 33.172 mm with minimum deformation at the board’s edge, as shown in Figure 12.

**Figure 13.**
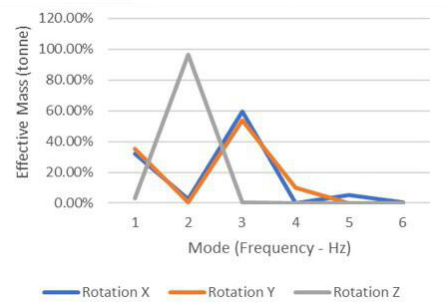
Effective mass to frequency for 8-member support.

As seen in Figure 13 and Table 3, more than 95% of the effective mass was the participating mode in the Z direction, 60% in the X direction, and slightly below 60% of the mode’s effective masses in the Y direction.

**Table 3.** Mass participation for 8-member support.

Mode	Rotation X	Rotation Y	Rotation Z
1	33.442	25.723	5.2324
2	2.67E+00	4.11E-01	1.53E+02
3	6.17E+01	3.95E+01	4.72E-01
4	6.09E-03	7.47E+00	2.01E-01
5	5.24E+00	1.06E-02	1.01E-03
6	3.00E-01	3.94E-03	8.40E-03
	103.3538	73.10313	158.784

**Figure 14.**
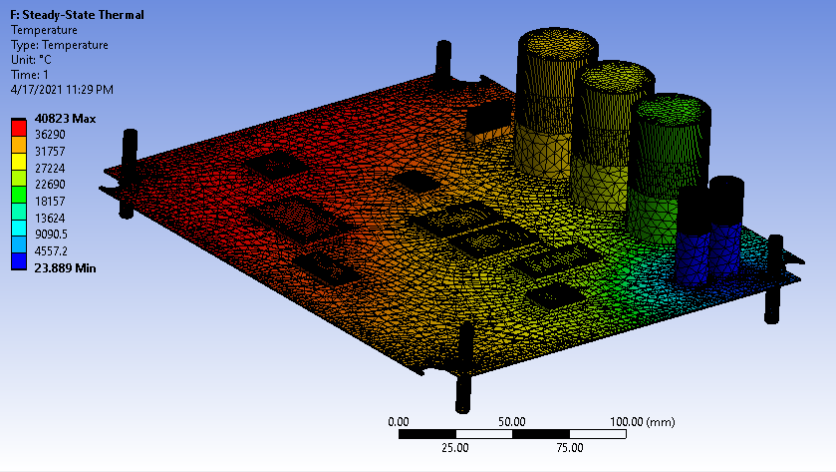
Thermal state of the circuit board.

The blue part of the circuit board shown in Figure 14 is the battery of the modeled circuit board, which is used to power the board. The temperature is distributed at this point, a critical factor that significantly impacts the performance of the entire circuit board. The heat dissipation to the circuit was high, more than 40,000 °C compared to the initial temperature of 23 °C based on the surface area and dissipation rate.

**Figure 15.**
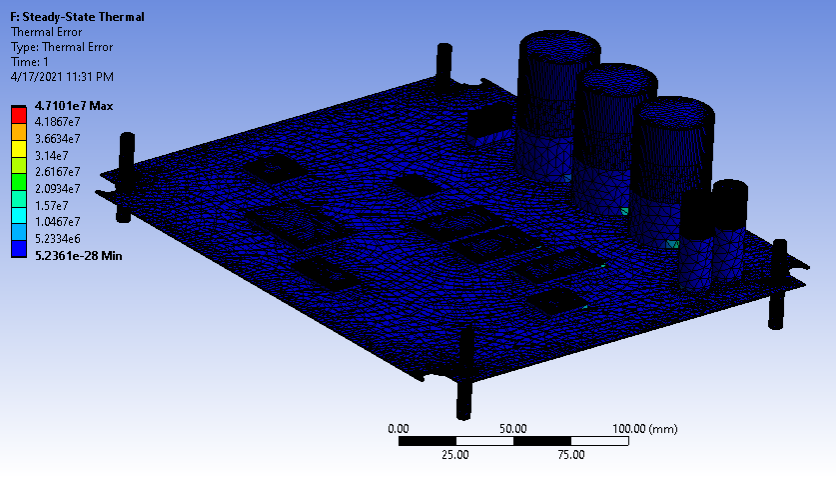
Thermal error of the circuit board.

The battery’s thermal error, as shown in Figure 15, is higher than expected. This is due to the temperature dissipated within the components encountered by the circuit board. The temperature effect gives a higher thermal error at a short time interval, which will lead to a rapid and concerning rate of the circuit board’s deformation.

## Discussion

### Principal Findings

This study made a unique contribution to the field of medical device design by employing FEA analysis to evaluate the modal and thermal responses of an AED. The use of Ansys Workbench for static structural, modal, and steady-state thermal analysis provided novel insights into the relationship between modal frequencies and thermal effects on the AED’s circuit board, focusing on component deformation and heat dissipation.

The FEA model demonstrated natural frequencies consistent with similar experimental models. This alignment indicates the robustness of the model but also highlights the influence of boundary conditions and the smeared property approach in the simulation. The boundary conditions may have contributed to the observed differences in frequency ranges between the FEA and experimental models, reflecting the inherent complexity of capturing real-world constraints within a simulation environment.

Overall, the findings underscore the crucial role of considering thermal and mechanical factors in the design of medical devices like AEDs, particularly for ensuring reliability under varying operational conditions. The implications of this study could significantly influence the field of medical device design, leading to the development of more robust and reliable AEDs. The study’s findings could shape the design and development of AEDs, resulting in devices that are better equipped to withstand the challenges of real-world use, thereby improving patient outcomes and enlightening the field.

### Strengths

First, including both prestressed and unprestressed modal analysis adds a layer of accuracy to the model, which improves the simpler FEA models used in previous research. The 0.0003% error difference in the prestressed analysis results is significant as it ensures the model’s accuracy, which is crucial for predicting AED performance. This level of precision is a substantial improvement over previous models, indicating the potential for more reliable AED designs.

The study’s ability to isolate the battery’s thermal effects and provide practical design recommendations, such as using separate boards or cooling mechanisms, equips engineers and researchers with actionable solutions for improving the durability of AEDs. These recommendations, such as using separate boards for the battery to reduce thermal stress on the main circuit board or implementing a cooling mechanism like a fan to manage the heat effectively, can be directly implemented in device designs, thereby enhancing the reliability of AEDs.

Lastly, the research leverages detailed meshing and refined convergence methods, ensuring uniformity in the results and yielding more reliable data for future applications in device design. This emphasis on methodological rigor should instill confidence in the audience about the reliability of the study’s data and its potential for future applications in device design.

### Comparison to Prior Work

The results are consistent with prior studies in the field, such as [10,11], which similarly demonstrated the adverse effects of vibrations and temperature fluctuations on medical devices like AEDs. However, this study extends previous findings by incorporating a prestressed analysis that improved natural frequency estimations, showing a minimal percentage error of 0.0003%. This precision in the FEA model instills confidence in the audience, representing an advancement in predicting deformation and fatigue, especially compared to earlier studies that primarily focused on random vibration fatigue or single-mode vibration analysis.

### Limitations

During this study, the detailed joints between the components and the circuit board are not considered; the emphasis is on the face-to-face contact of the components with the circuit board.

Second, the parametric iteration method used to determine the damping varies from 0.001 to 0.005 in a step of 0.005 sec. This limitation could lead to discrepancies between the model’s predictions and real-world outcomes, especially under varying temperatures and vibrational stresses that are not uniformly distributed in practice.

Third, while the study used prestressed analysis and provided accurate frequency improvements, the material properties of the components, such as capacitors and batteries, were modeled with assumptions that may not fully capture the complexity of real-world materials. For instance, variations in thermal expansion coefficients and conductivity might behave differently under prolonged or extreme use, leading to more pronounced deformations than simulated.

Lastly, the study did not extend its analysis to long-term fatigue testing of components under cyclic loading. Without fatigue life predictions, it is unclear how the PCB and its components would withstand continuous use over time, especially in emergency medical environments where reliability is critical.

### Conclusion

The conclusion of this study highlights the importance of prestressed analysis in improving the accuracy of vibration analysis for PCBs used in critical medical devices like AEDs. The presence of zero frequencies, which are attributed to the rigid body modes, introduces superfluous effects that were effectively minimized through weak springs, improving the overall accuracy of the vibration response. This step enhanced the natural frequency results, as shown by the prestressed analysis, which produced a negligible error of 0.0003%, demonstrating its precision.

The stiffness of the modeled circuit board was unevenly distributed due to the varying materials and components mounted on it, such as capacitors, which exhibited pronounced deformation. It is recommended to use flat capacitors of lower height to mitigate this issue, as they are less prone to deformation under vibration stress. Additionally, the significant heat dissipation from the lithium battery, which has a high specific heat capacity, was identified as a potential source of thermal stress, affecting the PCB’s long-term reliability. The study suggests using a dedicated cooling system, such as a fan, or placing the battery on a separate board to manage the heat effectively.

The findings suggest that the prestressed analysis method, enhanced thermal management strategies, and design modifications may improve the durability and performance of PCBs in real-world operating conditions. This could be important for the reliability of medical devices in environments like ambulances, where continuous vibration and thermal fluctuations occur.

## Abbreviations

AED: automated external defibrillator
CPR: cardiopulmonary resuscitation
EMS: emergency medical services
FEA: finite element analysis
OHCA: out-of-hospital cardiac arrest
PCB: printed circuit board
